# Neurology of the Cryopyrin-Associated Periodic Feversyndrome

**DOI:** 10.1186/1546-0096-13-S1-O36

**Published:** 2015-09-28

**Authors:** T Parker, S Keddie, D Kidd, M Maviki, T Lane, P Hawkins, L Ginsberg, H Lachmann

**Affiliations:** 1 University College London, Institute of Neurology, London, UK; 2 Royal Free Hospital London NHS Foundation Trust, Neurology, London, UK; 3 Royal Free Hospital London NHS Foundation Trust, Radiology, London, UK; 4 University College London, UK National Amyloidosis Centre, London, UK

## Introduction

There are little data on the multiple neurological manifestations of CAPS in adult patients.

## Objectives

To retrospectively analyse the neurological features of CAPS in a cohort of adult patients treated at a single UK centre.

## Patients and methods

38 patients (aged 16-69 years) with confirmed CAPS and on long term treatment with anti IL-1 agents were included. All patients were reviewed annually by a neurologist, an ophthalmologist and had audiometry. 35 patients had cranial MRIs, 4 patients had lumbar punctures.

## Results

Ninety-five percent of our patients had neurological features, with 84% describing some form of headache, 66% having sensorineural hearing loss on audiometry, 60% reporting myalgia, 34% having papilloedema during the course of their illness and 26% having evidence of optic atrophy. Twenty-five patients had normal MRI brain scans. Six scans demonstrated T2 hyperintensities in the subcortical white matter (in one case from prior ischaemic insult). Incidental findings in 4 cases included: an arachnoid cyst, an acoustic neuroma, a pineal cyst and a meningioma. All lumbar punctures were consistent with chronic meningitis. There was a marked response to pharmacological interleukin-one-beta (IL-1β) inhibition, (29/32 patients with headache and 22/23 with myalgia responding). Patients with the T348M mutation (N= 8) tended to have a more severe neurological phenotype with an earlier age of onset, more hearing loss, papilloedema and optic disc atrophy, the R260W mutation (N=8) was associated with an intermediate severity phenotype, and A439V (N=11) a relatively milder neurological phenotype.

## Conclusion

This case series demonstrates a much higher prevalence of neurological symptoms in CAPS than reported from EUROFEVERS (Levy 2014) and highlights the importance of increased awareness of CAPS amongst neurologists, to aid diagnosis and allow implementation of the highly targeted and effective therapies available.

